# The Book World of Medicine and Science

**Published:** 1895-02-23

**Authors:** 


### The Book World of Medicine and Science.

Organic Chemistry. By W. H. Perkin and F. Stanley Kipping. Part 2. Price 3s. 6d. (London: W. and R. Chambers. 1894.)

We had occasion some time since to notice the publication of the first part of the above work on organic chemistry. The high standard of lucidity and conciseness there initiated has been consistently maintained in this, the second part of the work. The principal subject matter of Part 2 is concerned with the properties of coal-tar and its derivatives. The study of these substances forms an excellent introduction to the whole question of aromatic compounds, and the individual members of the series have an important bearing on industrial questions and pharmaco-dynamical problems. So important are these compounds that it is impossible to grudge even a lavish expenditure of space in dealing with one or two of the fundamental points. The discussion of aniline dyes, including as it does such well-known substances as rosanaline, fluoresceïn, eosin, and the azo-dyes Bismarck brown, resorsin, and Congo-red, cannot fail to be of value to the student of physiology. The alkaloids have been dealt with rather from the aspect of therapeutic importance, than from the standpoint of chemical constitution. This, perhaps, is a mistake, since the subject is still in its infancy and difficult to grasp, even when regarded in a comprehensive and philosophic spirit. The exposition of a few isolated facts, and the stringing together of a heterogeneous collection of alkaloids is hardly the way to meet the difficulty. In most respects, however, this work fulfils its object as satisfactorily to the student as it does honour to the collaborateurs.

Kelly's London Medical Directory, 1895. (London: Kelly and Co.)

The present volume of Kelly's Directory maintains the character of this useful book of reference. The information, as in former years, relates to the medical schools, medical societies, and societies and boards in any way connected with the profession. The list of names, addresses, qualifications and literary works of the medical men in London includes those in the suburbs also—all most conveniently arranged for reference.

Our Dogs: A Weekly Journal devoted solely to Dogs. Price 1d.

This year seems to have opened with quite a boom in dogs; not only has a new society, "The Ladies' Kennel Association," sprung up, but we are able also to chronicle the appearance of a new journal, published under the above title. We were greatly interested in the reading of the first number, and believe that dog fanciers and lovers of dogs have now got a really first-class organ in which to express their views and find their requirements.

The Year-Book of Treatment, 1895. (London: Cassell and Co. Price 7s. 6d.)

The production of medical treatises is so continuous, and the flood of periodical literature so great, that the issue of occasional summaries giving the position attained by each branch of knowledge up to date becomes an absolute necessity. This we endeavour to provide for our readers, week by week, under the head of "Medical Progress," not giving a mere resumé of the week's literature, but so grouping the information, which is gathered together by experts in the several departments, that each class of subject shall be commented upon several times a year and the progress which has been made shall be recorded and discussed. This, we think, is the most useful plan, far better than that of giving snippets of news on every subject every week, and we believe that with the improved index which we propose to issue any practitioner who binds The Hospital will find himself independent of the various yearly summaries which are now issued. At the same time it is clear that a handy volume like the now well-known "Year-Book of Treatment," is extremely useful as a book of reference, its restriction to treatment alone and the absence of those more or less ephemeral comments on passing events which make a weekly journal interesting, rendering the "Year-Book" a pleasant companion and a convenient storehouse of information. Among the great number of subjects treated it would be both difficult and invidious to select any for special mention, in fact, one of the things which deserves the greatest commendation in regard to this book is that the balance between the various matters treated on is so well preserved that no one article stands out prominently among the rest.

